# Effects of Calcium on Arsenate Adsorption and Arsenate/Iron Bioreduction of Ferrihydrite in Stimulated Groundwater

**DOI:** 10.3390/ijerph19063465

**Published:** 2022-03-15

**Authors:** Mengna Chen, Zuoming Xie, Yang Yang, Ban Gao, Jia Wang

**Affiliations:** 1 Hubei Key Laboratory of Yangtze Catchment Environmental Aquatic Science, School of Environmental Studies, China University of Geosciences, Wuhan 430074, China; chenmengna0202@163.com (M.C.); 15072815217@cug.edu.cn (Y.Y.); jiawang_cug@163.com (J.W.); 2 State Key Laboratory of Biogeology and Environmental Geology, China University of Geosciences, Wuhan 430074, China; 3 State Key Laboratory of Freshwater Ecology and Biotechnology, Institute of Hydrobiology, Chinese Academy of Sciences, Wuhan 430072, China; bangao@cug.edu.cn

**Keywords:** ferrihydrite, calcium, electrostatic adsorption, As bioreduction, groundwater

## Abstract

The reduction and transformation of arsenic-bearing ferrihydrite by arsenate-iron reducing bacteria is one of the main sources of arsenic enrichment in groundwater. During this process the coexistence cations may have a considerable effect. However, the ionic radius of calcium is larger than that of iron and shows a low affinity for ferrihydrite, and the effect of coexisting calcium on the migration and release of arsenic in arsenic-bearing ferrihydrite remains unclear. This study mainly explored the influence of adsorbed Ca^2+^ on strain JH012-1-mediated migration and release of arsenate in a simulated groundwater environment, in which 3 mM ferrihydrite and pH 7.5. Ca^2+^ were pre-absorbed on As(V)-containing ferrihydrite with a As:Fe ratio of 0.2. Solid samples were analyzed by X-ray diffraction (XRD), scanning electron microscopic (SEM), Fourier transform infrared spectroscopy (FTIR), and X-ray photoelectron spectroscopy (XPS). The results show that calcium and arsenate can synergistically adsorb on ferrihydrite due to the electrostatic interactions, and the adsorbed Ca^2+^ mainly exists on the surface through the outer-sphere complex. Adsorbed Ca^2+^ entering the stimulated groundwater was easily disturbed and led to an extra release of 3.5 mg/L arsenic in the early stage. Moreover, adsorbed Ca^2+^ inhibited biogenic ferrous ions from accumulating on ferrihydrite. As a result, only 12.30% Fe(II) existed in the solid phase, whereas 29.35% existed without Ca^2+^ adsorption. Thus, the generation of parasymplesite was inhibited, which is not conducive to the immobilization of arsenic in groundwater.

## 1. Introduction

Arsenic is a poisonous metalloid element occurring in various environments. A series of public health accidents due to arseniasis have been reported around the world [1,2,3]. Arsenic enrichment in the aquifers of Bangladesh and the Mekong Delta caused by a combination of geochemical and microbial processes has been thoroughly studied [4,5]. In addition, in groundwater of West Johannesburg, arsenic emissions reached 6150 μg/L from highly mineralized rocks including arsenopyrite (FeAsS), arsenical oxide, sulpharsenide, arsenopyritical reefs, leucopyrite, löllingite (FeAs_2_) and scorodite (FeAsO_4_·2H_2_O) [6].

Poorly ordered 2-line ferrihydrite (Fh) with high surface reactivity is ubiquitously distributed in the natural environment [7], and is regarded as an important sink for nutrients and contaminants including arsenic [8]. However, metastable ferrihydrite can transform to more crystalline iron (oxyhydr)-oxides spontaneously, thus affecting the fate of arsenic through adsorption/desorption [9] and precipitation/dissolution [10,11]. The transformation of ferrihydrite under aerobic and neutral pH conditions is very slow, usually taking from months to years [12], whereas the transformation by dissimilatory iron-reducing bacteria (DIRB) only needs several hours or days in anaerobic and non-sulfidic environments [13]. Thus, DIRB can indirectly promote the release or sequestration of arsenic binding with iron-containing minerals. Dissimilatory arsenate-respiring prokaryotes are also an important bacteria involved in the cycle of arsenic, because they can obtain energy by metabolizing arsenate while converting it to trivalent arsenic [14,15,16]. Due to the high mobility of arsenite and the strong correlation between arsenic and iron (oxyhydr)-oxides, bacteria possessing both arsenate- and iron-reducing capacity play a significant role in the migration and transformation of arsenic in groundwater [17,18].

Calcium is a widely present cation in groundwater [19]. Previous research about the role of calcium in the migration of arsenic under neutral conditions has mainly focused on simple adsorption by calcium minerals [20,21]; the adsorption of microbiologically induced carbonate precipitation in a highly alkaline groundwater environment [22]; and increasing the sorption capacity of arsenic through the formation of Fe-OM-Ca ternary complexation [23]. Both of these processes lead to arsenic removal. Unlike cations such as Al^3+^, Mn^2+^/Mn^3+^/Mn^4+^, Zn^2+^ and Cd^2+^ [24,25,26,27], the effects of Ca^2+^ alone on the mobilization of arsenic in iron (oxyhydr)-oxides have been rarely studied. This may because the physicochemical properties of calcium, including binding ability constants and ionic radii, limit both the adsorption and substitution of the structure of calcium on ferrihydrite [28,29]. Antelo et al. [30] found that Ca^2+^ can promote the adsorption of arsenate on ferrihydrite by electrostatic adsorption when the pH value is above 8. In the suboxic environments involving iron reduction, reductive dissolution of scorodite may cause the release of a large amount of arsenic [31], reaching about 115 mg/L [32]. The dissolved iron ions form ferrihydrite under neutral groundwater conditions and absorb large amounts of arsenic. In this case, the influence of adsorbed Ca^2+^ on the fate of arsenic associated with ferrihydrite is particularly important.

Therefore, ferrihydrite-adsorbed calcium and arsenate were synthesized under neutral pH conditions using a As/Fe ratio of 0.2 and subsequently incubated with strain JH012-1 in stimulated groundwater conditions. This study aimed to clarify: (1) the effect of Ca^2+^ on the adsorption of arsenate in ferrihydrite under neutral pH; (2) the influence of pre-adsorbed calcium on the biotransformation of arsenate-bearing ferrihydrite; and (3) the biorelease and migration of arsenate in arsenate-bearing ferrihydrite in the presence and absence of adsorbed calcium in a simulated groundwater system.

## 2. Materials and Methods

### 2.1. Bacterial Incubation

*Citrobacter* sp. JH012-1 was isolated by our group from the aquifer of Shayang country in Hubei province, and contains both arrA and arsC genes, and possesses an iron-reduction ability. *Citrobacter* sp. JH012-1 has the gene bank accession number MZ227386 of SUB9679906 citrobacter. Before each experiment, JH012-1 was cultured in classical LB medium for 24 h. After washing three times, batch experiments with 5% bacterial suspension were conducted in anaerobic minimal salt medium (MSM) (0.1 g/L NH_4_Cl, 0.5 g/L KCl, 1 g/L NaCl, 0.027 mg/L KH_2_PO_4_, 0.5 g/L yeast extract and 0.6 g/L glucose), and the pH of the solution was buffered to 7.5 by 30 mM Bis-Tris. Briefly, 200 mL MSM medium was transferred to 250 mL anaerobic bottles; after purging with ultrapure N_2_ for over 30 min, 2 mL concentrated cell cultures was injected into the bottles. Finally, all of the reactors were sealed with Teflon-coated butyl rubber stoppers wrapped in Al foil, and placed on a 200 r/min shaker and cultured at 32 °C.

### 2.2. Synthesis of Arsenate and Mutual Arsenate–Calcium-Adsorbed Ferrihydrite

Pure ferrihydrite material (Fh) was prepared according to Xiao [33] with slight modification. Briefly, 0.01 M FeCl_3_ solution was adjusted to pH 7.5 with 2 M NaOH under continuous stirring. The ferrihydrite dispersion was then aged at room temperature for 16 h and washed with deionized water to reduce the conductivity to about 50 μS/cm. The arsenate-adsorbed ferrihydrite (As-Fh) was prepared by shaking the mixture of 10 mM ferrihydrite and 2 mM Na_3_AsO_4_·12H_2_O at 200 r/min in room temperature. For arsenate–calcium-adsorbed ferrihydrite (As-Ca-Fh), 10 mM ferrihydrite was pre-equilibrated with 5 mM CaCl_2_ solution for 30 min, and then Na_3_AsO_4_·12H_2_O was added to reach the concentration of 2 mM. The adsorption reaction lasted for 96 h; during this process, the solution pH was maintained at 7.5 by adding 1 M NaOH or 1 M HCl.

### 2.3. Arsenate Adsorption Experiment

To explore the influence of dissolved Ca^2+^ on the adsorption of arsenate on ferrihydrite, adsorption experiments were performed in 50 mL centrifuge tubes, and stirred at room temperature at 200 r/min for 96 h. Solution pH was adjusted to a range of 4–9 with 1 M NaOH or HCl solution in 0.5 pH increments. Specifically, 10 mM ferrihydrite and 2 mM arsenate and 5 mM calcium were used to trigger the adsorption. The suspension was filtered through Nalgene acetate membrane (pore size < 0.22 µm), and the residual arsenate concentration in the filtrate was measured. The amount of arsenate adsorbed on ferrihydrite was assessed by the difference between the initial and the final concentrations. Ultrapure water was used in all experiments.

### 2.4. Bioreduction of Ferrihydrite

Biotic reduction and transformation for Fh, As-Fh and As-Ca-Fh at a 3 mM dosage were investigated. The reaction was performed in a 250 mL anaerobic bottle containing 200 mL MSM medium. All experiments were conducted in triplicate. Samples were taken anoxically after inoculation with strain JH012-1. For each time point, three parallel bottles were sampled and analyzed for total and dissolved Fe(II) and Fe(III). The other part of the samples was frozen at −20 °C until measurement of total dissolved As and inorganic arsenic speciation. Since As(III) and As(V) were separated immediately, the operation of freezing the samples did not affect the experimental results. The minerals were harvested in a glovebox through decanting the supernatant followed by centrifugation at 5000 r/min for 10 min and vacuum drying of the mineral pellet. The mineral powder of three parallel bottles was collected for mineral analysis.

### 2.5. Chemical Analyses

Total and dissolved Fe(II) and Fe(III) were quantified spectrophotometrically (UV-1800PC, Shanghai Mapada Instruments Co., Ltd., Shanghai, China) using the ferrozine assay according to Stookey [34], from which Fe(II) was directly quantified with the ferrozine assay, and Fe(III) was analyzed by 2.5% ascorbic acid containing ferrozine solution. As(V) and As(III) were separated by a silica-based strong anion-exchange cartridge according to [35]. Total arsenic, As(III) and As(V) were then measured by AFS (HG-AFS; AFS-830, Beijing Jitian Instrument Co., Ltd., Beijing, China). Aliquots for aqueous-phase analysis were passed through 0.22 μm filters. The total arsenic and iron in initial ferrihydrite were obtained by the aquaregia digestion method and the solid associative Fe(II) was extracted by 1 M HCl solution [36]. The pH was measured by a pH meter.

### 2.6. Mineralogical and Spectroscopic Analyses

The crystal structure was determined by a powder X-ray diffractometer (Bruker D8 Advance). Cobalt radiation was used at 40 kV and 40 mA over a scan range of 10~90° 2θ on a copper target. Crystalline materials were identified using MDI jade 6 software. Fourier transform infrared spectroscopy was carried out by Nicolet 6700 FTIR spectrometer (Thermo Fisher, Waltham, MA, USA) at lower than 0.09 cm^−1^ resolution in transmission mode in the range of 4000–400 cm^−1^ and analyzed by OMNIC software. Selected minerals were examined by scanning electron microscopy-energy dispersive X-ray spectroscopy (SEM-EDX) using a ZEISS Gemini 300 scanning electron microscope with EDX. Samples for SEM examination were pasted onto aluminum stubs using double-sided carbon tape. A thin-layer gold-coated sample was prepared using the ion sputter technique. Mapping was undertaken at a working distance of 8.5 mm. The binding energies and atomic ratios of arsenic on the surface of the materials were analyzed by X-ray photoelectron spectroscopy (XPS) on a ESCALAB Xi+ (Thermo Fischer) equipped with a rotating Al anode. The working voltage was 12.5 kV and the filament current was 16 mA. The passing energy for both full spectrum and narrow spectrum were 100 and 20 eV, respectively. C1s at 284.8 eV binding energy was used as the energy standard for charging correction.

### 2.7. Surface Complexation Modeling

The three-plane charge distribution surface complexation model (CD-SCM) for ferrihydrite established by Tiberg et al. was used in this study [37]. In this model, the ferrihydrite surface area is 650 m^2^/g with an inner capacitance of 1.15 F/m^2^, an outer capacitance of 0.9 F/m^2^, and a site density of 7.8 sites/nm^2^. The reactive ferrihydrite surface groups contain both singly (≡FeOH^½−^) and triply (≡Fe_3_O^½−^) coordinated surface groups, but only the former is reactive to surface complexes. The surface complexation reactions and the logK values used in the CD-MUSIC model are given in Table 1 and were obtained from [30] and [37,38]. The CO_2_ pressure was considered to be 0.00038 atm. The surface arsenate and calcium speciation were then calculated with Visual MINTEQ 3.1.

## 3. Results

### 3.1. Impacts of Arsenate and Calcium Adsorb on Ferrihydrite Spectral Signature

**Figure 1:**
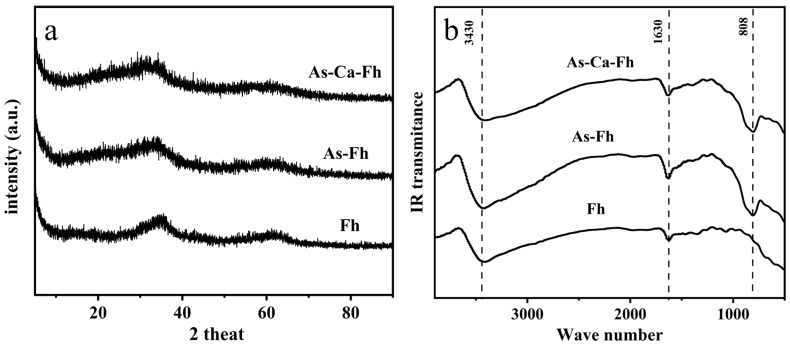
Initial XRD patterns (**a**) and FTIR spectra (**b**) of Fh, As-Fh, and As-Ca-Fh.

**Figure 2:**
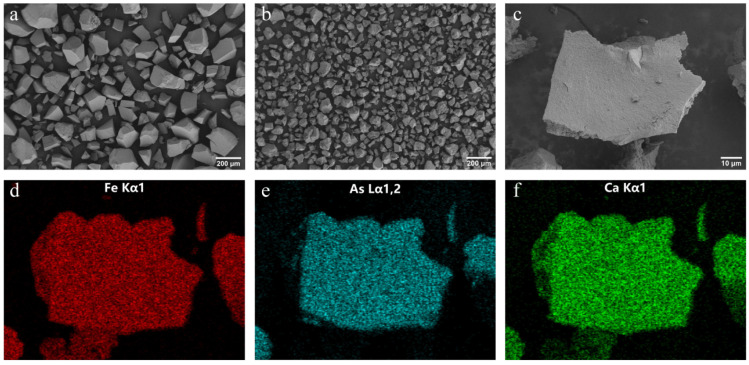
SEM morphology image of As-Fh (**a**) and As-Ca-Fh (**b**) before bioreaction. Selected EDS mapping areas (**c**) and elemental distribution of Fe (**d**), As (**e**), and Ca (**f**) of As-Ca-Fh.

To determine whether the adsorption of As and Ca can alter the structure of ferrihydrite, X-ray diffraction, Fourier transform infrared spectroscopy, and scanning electron microscopy were conducted. X-ray diffraction (Figure 1a) analysis identified only two characteristic peaks of 2-line ferrihydrite, suggesting that no arsenic- or calcium-bearing mineral were formed. The spectral identification for Fh, Fh-As, and Fh-As-Ca was shown in FTIR spectra (Figure 1b). The band at 3430 cm^−1^ was associated with the surface hydroxyl group [39]. The bands at 808 cm^−1^ and 1630 cm^−1^ were attributed to adsorbed arsenate and surface water, respectively [40], whereas the bands of Ca-As and Ca-O in the low frequency region (<900 cm^−1^) were not observed, indicating the arsenate-bridged ternary complexes were not formed [41]. The morphology of Fh-As and Fh-As-Ca pre-biotransformation examined by SEM (Figure 2a,b) showed that both of these are amorphous shapes with smooth surfaces. EDS mapping (Figure 2c) was used to analyze the related element composition and spatial distribution of the micromorphological properties of As-Ca-Fh. It proved that As (Figure 2e) and Ca (Figure 2f) were homogeneously distributed on the ferrihydrite surface (Figure 2d), and further verified that the surface precipitation of Ca-As(V) and Ca-O was not formed during the arsenate adsorption on ferrihydrite in the presence of Ca^2+^. The As/Fe atomic ratios were 10.4% and 16.9% in As-Fh and As-Ca-Fh (Table 2), respectively, indicating an increase in arsenate adsorption with the presence of calcium.

### 3.2. Mechanism of Calcium Promotes the Adsorption of Arsenate on Ferrihydrite

**Figure 3:**
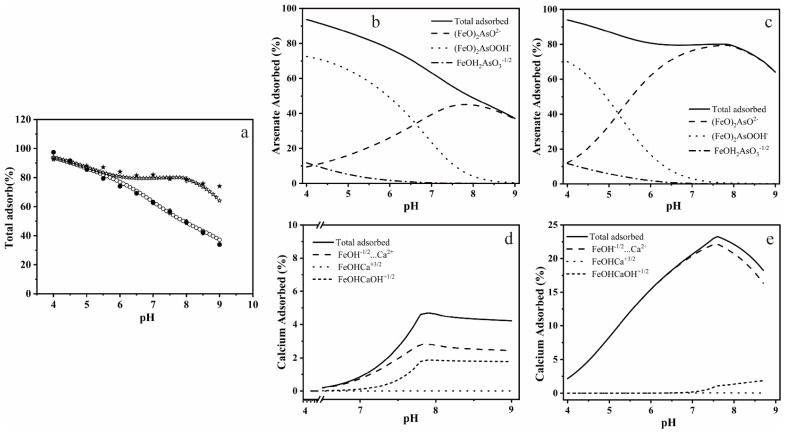
Arsenate adsorption on ferrihydrite as a function of pH. The pentagram and circle shapes indicate arsenate adsorption in the presence (☆ for simulated data, ★ for measured data) and absence (○ for simulated data, ● for measured data) of 5 mM Ca^2+^, respectively (**a**). The surface speciation distribution of As and Ca on ferrihydrite as a function of pH with (**c**,**e**) or without (**b**,**d**) the presence of Ca^2+^.

Figure 3a shows the arsenate adsorption envelopes in the absence and presence of calcium for an initial concentration of As, Fe, and Ca mentioned in Section 2.3. Figure 3b–e shows the arsenate and calcium surface speciation as a function of pH according to the modeling calculations. Adsorption of arsenate decreased gradually and continuously as the pH increased in the absence of calcium, which was consistent with previous studies [42]. In the presence of Ca^2+^, the change in arsenic adsorption was negligible at pH < 6.0 and, as the pH increased from 6 to 9, a slow decline in arsenate adsorption from 80.71% to 64.12%, compared with 76.95% to 37.18% in the absence of calcium, was observed. The extra adsorption of arsenate in the calcium-containing treatment was mainly attributed to non-protonated bidentate surface complexes. The same simulation method was used to compare the adsorption of Ca^2+^ on ferrihydrite alone with that on arsenate adsorption ferrihydrite. The results showed that arsenate also promoted the adsorption of Ca^2+^, and the adsorption mode of Ca^2+^ was mainly on the outer-sphere complex, which is consistent with the Ca^2+^ adsorption on arsenate-containing goethite [43].

### 3.3. Bioreduction and Transformation of Ferrihydrite

**Figure 4:**
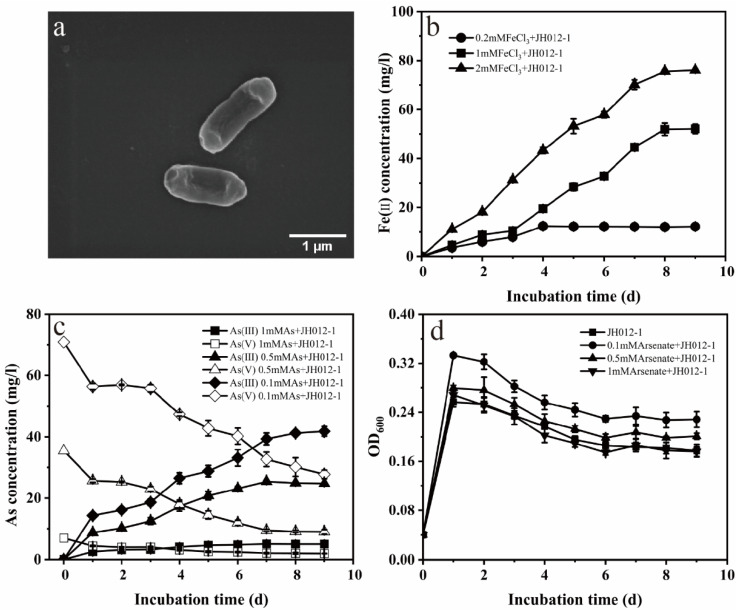
Morphology of strain JH012-1 (**a**). Iron (**b**) and arsenate (**c**) reduction ability of JH012-1; growth of JH012-1 under different concentrations of arsenate (**d**).

**Figure 5:**
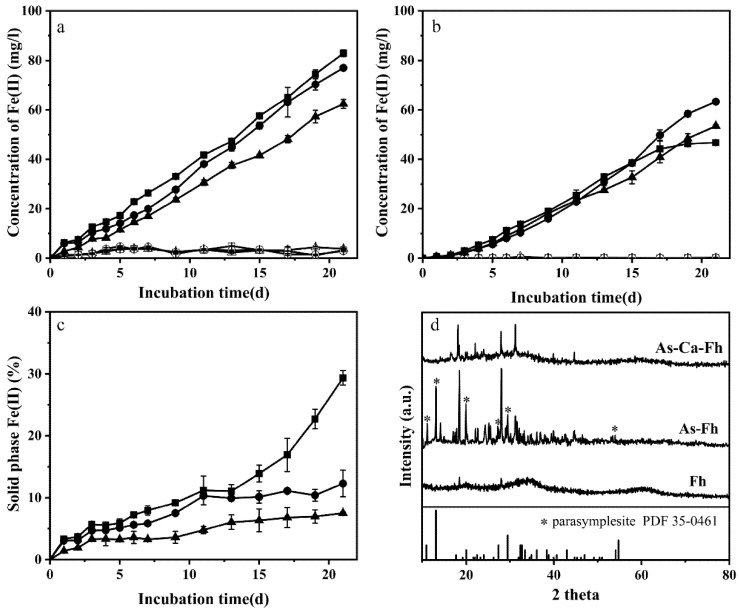
Measured total Fe(II) concentrations (**a**), solution Fe(II) concentration (**b**), proportion of Fe(II) in the solid phase (**c**), and XRD patterns of post-reaction ferrihydrite * stands for parasymplesite (**d**). Triangles ▲, circles ●, and squares ■ represent Fh, As-Fh, and As-Ca-Fh, respectively, and the corresponding hollow shape represents sterile treatment.

The Fe(III) and As (V) reduction capacity of JH012-1, and the effect of different arsenate content on bacteria growth, can be seen in Figure 4. Arsenate concentration below 0.5 mM can promote the growth of JH012-1; however, when the arsenate concentration reached 1 mM, the promotion effect disappeared (Figure 4d). In addition, JH012-1 can completely reduce Fe(III) below 1 mM within 8 days (Figure 4b). Studies on the reduction of arsenate show that the rate and amount of arsenate reduction will increase with the increase in arsenate concentration (Figure 4c). The bioreduction and transformation of Fh, As-Fh, and As-Ca-Fh is shown in Figure 5. The total Fe(II) content (Figure 5a) reflected that the bioreduction of As-Fh and As-Ca-Fh by JH012-1 was better than that of Fh, and the mutual adsorption of Ca^2+^ had little effect on the reduction of ferrihydrite. In contrast, the total amount of Fe(II) in As-Fh increased steadily during the whole experiment, while the rate of increase of Fe(II) in the solution declined gradually after 13d (Figure 5b). To further analyze the proportion of Fe(II) on the solid phase, we calculated the ratio between the solid Fe(II) (total HCl extractable Fe(II) minus solution Fe(II) and the remaining solid phase iron content (total iron content minus solution iron content) (Figure 5c). It was found that the proportion of solid Fe(II) in the As-Fh group showed a continuous accumulation and an obvious increase after 13 days, reaching 29.35% at the end of the reaction. By comparison, in the As-Ca-Fh group, it remained steady after 13 days, but the values for both groups were higher than that in Fh.

**Figure 6:**
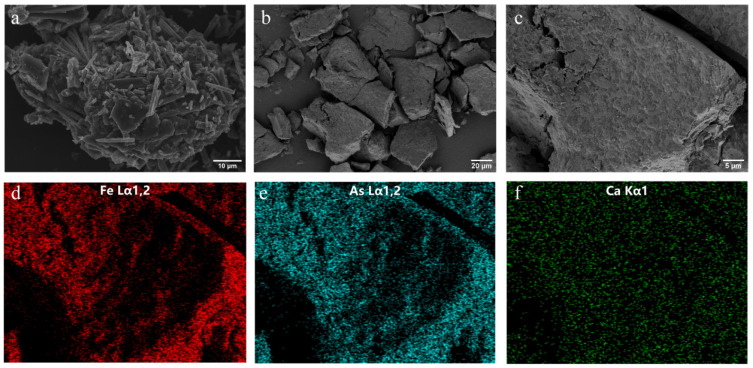
SEM morphology image of As-Fh (**a**) and As-Ca-Fh (**b**) after bioreaction; selected EDS mapping areas (**c**) and elemental distribution of Fe (**d**), As (**e**), and Ca (**f**) of As-Ca-Fh.

The bioreduction products were characterized (Figure 5d). The peak pattern of As-Fh and As-Ca-Fh changed significantly, and As-Fh possessed the characteristic peak of parasymplesite. Unfortunately, it is impossible to judge the mineral types represented by other diffraction peaks because of the disorderly peak type of ferrihydrite itself. Further analysis of the mineral surface for As-Fh by SEM showed that strip minerals were formed (Figure 6a), resembling the parasymplesite reported by [31]. No other minerals with special morphologies were found in As-Ca-Fh; EDS mapping of As-Ca-Fh showed that the mineral surface contained Fe, As, and Ca (Figure 6d–f), whereas the calcium content was only about 13.28% of the initial mineral (Table 2).

### 3.4. Bioreduction and Migration of Arsenic

**Figure 7:**
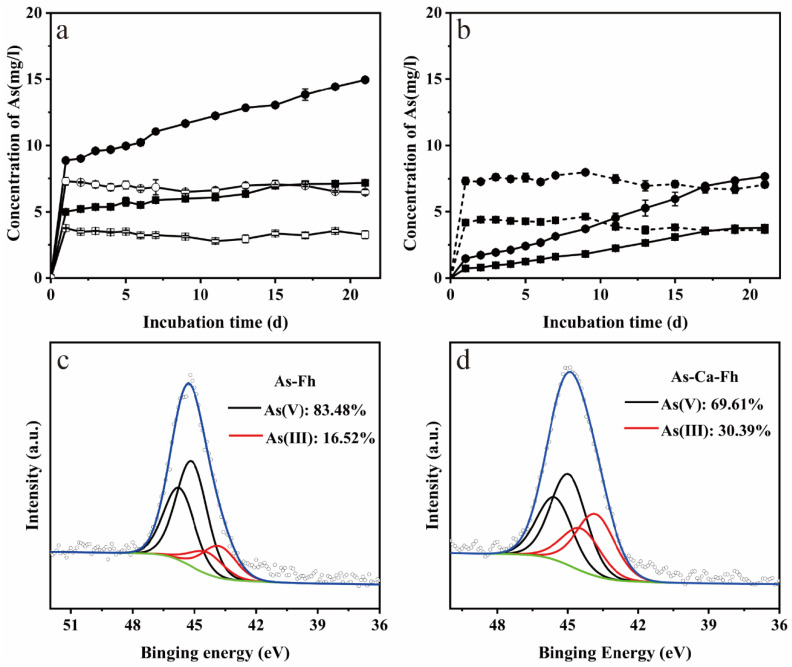
Measured total As (**a**), As(III) (solid line), and As(V) (dotted line) concentrations (**b**); XPS analyses of As species in the surface region of As-Fh (**c**) and As-Ca-Fh (**d**) where the blue and green color lines stands for photoelectron spectral lines of arsenic and the baseline. Squares ■ and circles ● in picture (**a**) and (**b**) represent As-Fh and As-Ca-Fh, respectively; the corresponding hollow shape in picture (**a**) (□,○) represents sterile treatment. The hollow shape in (**c**,**d**) means the same thing as the blue color line in it (the photoelectron spectral of arsenic). The difference is that hollow shape circles stand for the data point and the blue color line is the fitted line for hollow shape circles.

With the incubation of strain JH012-1, total aqueous As concentrations reached the concentrations of 5.0 and 8.8 mg/L, respectively, in As-Fh and As-Ca-Fh; compared to As-Ca-Fh, As release in As-Fh was much slower (Figure 7a). Similar to total As, release of As(III) in As-Ca-Fh on the first day was more rapid, and an increasing gap in As(III) concentration between As-Fh and As-Ca-Fh appeared over time while the concentration of As(V) remained stable during the whole process (Figure 7b). XPS measurements were taken to determine the speciation and relative percentages of arsenic in the near surface region. Analyses showed that As(V) was still the primary valence state. The qualitative analysis of the As(V) ratio in the solid phase suggested a higher proportion of 83.48% As(V) in As-Fh compared to 69.61% in As-Ca-Fh (Figure 7c,d).

## 4. Discussion

The relationship between the adsorption of calcium and arsenate on ferrihydrite and the subsequent bioreduction and transformation were studied. Synergistic adsorption of calcium and arsenate on ferrihydrite were observed. As analyzed in Figure 1, calcium neither changed the crystal structure of ferrihydrite nor formed Ca-O-As and Ca-O-Fe bonds in the low frequency region (<900 cm^−1^). The main reasons for this are as follows: (1) The ionic radius of calcium is larger than that of iron, and thus could not incorporated into its lattice structure; (2) due to the low binding constants for calcium towards ferrihydrite, it is not easy to form Ca-O-Fe bonds [44]; (3) the pH value range of calcium arsenate precipitation (pH 7.9–13.4) is higher than the pH of 7.5 used in this experiment [16]. In spite of this, in As-Ca-Fh, Ca^2+^ was still uniformly adsorbed on the surface of the ferrihydrite (Figure 2f) with a Ca/Fe molar ratio of 0.128 (Table 2), which was mainly due to the electrostatic interaction between As(V) and Ca^2+^ on the surface of ferrihydrite. For the same reason, the presence of Ca^2+^ can promote the adsorption of arsenate (Figure 3a). This phenomenon can be explained by the surface charge of ferrihydrite. The lower As(V) adsorption with increasing pH is often attributed to the reduction in positively charged surface species. The point of zero charge (PZC) for ferrihydrite ranges from 6–8 [45,46]; however, the adsorption of arsenate and other oxygen anions onto ferrihydrite can result in an increased negative charge on the surface of ferrihydrite, and thus a decreased PZC. Under this condition, the electrostatic repulsion between ferrihydrite and the negatively charged arsenate ions in the solution weakened the adsorption of arsenate [47]. As a consequence, the lower As(V) adsorption with increasing pH was observed. A large number of studies into calcium promoting the adsorption of arsenate and phosphate in ferrihydrite have suggested that Ca^2+^ can reduce this electrostatic repulsion, and thus promote the adsorption of arsenate or phosphate [30,48]. In this study, we used the three-plane charge distribution surface complexation model to verify the adsorption mechanism. It was found that, at pH 7.5, the majority of positive charges of the adsorbed Ca^2+^ located in the 1-plane can lower the electrostatic repulsion [49]. The extra arsenate adsorption in As-Ca-Fh was mainly in the form of bidentate complexes (Figure 3c), which retained more charges on the inner face compared to monodentate complexes, thus lowering the electrostatic repulsion in the 1-plane [50]. In addition, adsorbed As(V) significantly promotes the amount of Ca^2+^ adsorbed on the surface of ferrihydrite through the outer-sphere complex (Figure 3e). Therefore, it was considered in this study that Ca^2+^ and As(V) can synergistically adsorb on the ferrihydrite due to the electrostatic interactions. Unlike previous studies, adsorption can be promoted at near neutral pH, which may be due to the higher arsenate content in ferrihydrite.

In the incubations with JH012-1, reductive dissolution was the main cause of iron release. In addition, both As(V) and Fe(III) were simultaneously reduced for As-Fh and As-Ca-Fh, and this was thermodynamically reasonable because reduction of amorphous Fe(III)(hydroxide) oxides, such as ferrihydrite, yields a similar Gibbs free energy as in the reduction of As(V) [51]. Studies with Shewanella putrefaciens CN-32 suggested that ferrihydrite reduction rates decreased with increasing phosphate concentrations due to the phosphate surface coverage [52]. In theory, the presence of arsenate should have the same negative impact because of the similar adsorption properties of phosphate and arsenate on ferrihydrite [53]. However, in this biological reduction experiment, adsorbed arsenate promoted the reduction of ferrihydrite (Figure 5a). Campbell et al. previously reported that As(V) adsorbed onto the surface of ferrihydrite can enhance the rate of microbial Fe-(III) reduction [51]. The reason given was that the adsorbed As(III) reduced by Shewanella sp. ANA-3 may change the surface area and crystallinity of the oxide mineral. In addition, gene expression analysis of mtrDEF, omcA, and mtrCAB (the gene cluster involved in iron reduction) during arsenate and iron reduction in Shewanella sp. ANA-3 was undertaken [54]. The authors found that both insoluble and soluble Fe(III), in addition to arsenate and fumarate, can cause increased expression of omcA and mtrCAB. Compared with the wild-type ANA-3, the Fe(III) reduction of arsenate reduction mutant was in half less when arsenate was adsorbed in ferrihydrite. Since JH012-1 can reduce both As(V) and Fe(III), it is speculated that the As(V)-Fe(III) reduction has a similar relationship as that of ANA-3. In addition, in the early stage of the experiment in this study, the arsenate and iron reduction ability onJH012-1 was examined (Figure 4d). The result suggested that, when arsenate of 0.1 and 0.5 mM was added to the culture medium, the growth of JH012-1 was promoted. This may also be the reason for the promoted reduction of ferric iron.

The content of solid phase Fe(II) is an important index for the formation of ferrous minerals. Parasymplesite is the arsenate analog of vivianite having the formula Fe(II)_3_(As(V)O_4_)_2_·8H_2_O [11]. The formation of parasymplesite can be caused by the precipitation of dissolved Fe(II) and As(V), or by the adsorption of dissolved Fe(II) on the surface of the adsorbent complexed with As(V) [55,56]. In this experiment, parasymplesite was found only in the biological reduction products of As-Fh. Since no significant loss of As(V) was observed in solution, the formation of parasymplesite should be due to the precipitation of Fe(II) on the surface of arsenate-bearing ferrihydrite. Briefly, with the release of Fe(II) after the first day, the proportion of arsenate in As-Fh increased relatively. The Fe(II) in the solution started to complex with As(V) when it was sufficient for parasymplesite formation. The substantial increase in solid Fe(II) in the late stage of the reaction (Figure 5c) and relatively high proportion of As(V) on the surface of As-Fh observed by XPS support this (Figure 7c,d). In comparison, adsorbed Ca^2+^ inhibited the production of parasymplesite and the immobilization of arsenate. A large number of studies have concerned about the inhibition of the precipitation of Fe(II) by adsorbed cations [44,57]. Ca^2+^ in solution may also have an adverse effect on the re-adsorption of Fe(II) by competing for the adsorption sites [58,59].

Adsorbed arsenate was released violently on the first day (Figure 7a), since phosphate and organic components in the medium may have displaced part of As(V) from the surface of Fe(III)(hydroxide) oxides by competing for the same adsorption sites as As(V) [60]. Interestingly, As-Ca-Fh released more As(V) into the solution on the first day compared with As-Fh. The surface complexation model proved that calcium was mainly adsorbed through the outer-sphere complex in this study, which is close to the bulk solution and easily disturbed by the solution environment [61]. When transferred to a complex groundwater environment, parts of the Ca^2+^ were released into the solution, which was demonstrated by the reduced surface content of Ca^2+^ on As-Ca-Fh (Table 2). Consequently, the positive charge on the mineral surface was reduced and the electrostatic adsorption effect was weakened. For this reason, more As(V) was released into the solution. According to previous studies, the reduction kinetics of arsenate adsorbed onto ferrihydrite is predominately controlled by the availability of dissolved arsenate [62], and the expression of As(V)-reducing genes in arsenate-ferrihydrite systems is higher at high As/Fe ratios [63]. In our experiment, As-Ca-Fh possessed more arsenate, and the release of arsenate reached a higher layer in the first day than As-Fh; thus, the bioreduction of arsenate is relatively high at the beginning. Continuous observation showed that the release of arsenic and iron was not proportional, and the release of arsenic corresponded to the production of As(III). Therefore, it is speculated that the main reason for the release of arsenic after the first day was the reduction of adsorbed arsenate by JH012-1. Since the parasymplesite is crystalline and a less favorable form for microbial reduction [62], this weakens the reduction of arsenate by JH012-1. Therefore, at the end of the reaction, relatively small amounts, of 3.79 mg/L dissolved and 16.52% solid bonded As(III) (Figure 7) in As-Fh, were observed.

## 5. Conclusions

In this study, we demonstrate that calcium can obviously promote the adsorption of arsenate on ferrihydrite through electrostatic interaction under neutral pH conditions. However, when the mutual arsenate-calcium adsorbed on ferrihydrite is placed in a stimulated anoxic groundwater environment in the presence of strain JH012-1, a large proportion of adsorbed calcium is released into the solution. Nonetheless, the precipitation of biogenic ferrous ions on As-Ca-Fh and the formation of the crystalline ferrous arsenate mineral parasymplesite were still inhibited. This eventually leads to the mobilization of arsenic under anoxic conditions in the presence of JH012-1. In contrast to the immobilization of arsenic in highly alkaline groundwater containing calcium minerals, this paper may provide new insights into the effect of calcium-containing iron minerals on the biological migration and transformation of arsenic in a groundwater environment.

## Figures and Tables

**Table 1:** Surface species and CD model parameters for H^+^, AsO_4_^3-^, and Ca^2+^ binding to ferrihydrite.

Surfuce	≡FeOH	≡Fe_3_O	ΔZ_0_	ΔZ_1_	ΔZ_2_	H+	AsO_4_^3−^	Ca^2+^	logK
≡FeOH^−1/2^	1	0	0	0	0	0	0	0	0
≡Fe_3_O^−1/2^	0	1	0	0	0	0	0	0	0
≡FeOH^+1/2^	1	0	1	0	0	1	0	0	8.1
≡Fe_3_OH^+1/2^	0	1	1	0	0	1	0	0	8.1
≡(FeO)_2_AsO^−2^	2	0	0.47	−1.47	0	2	1	0	27.4
≡(FeO)_2_AsOOH^-^	2	0	0.58	−0.58	0	3	1	0	32.04
≡FeOH_2_AsO_3_	1	0	0.5	−0.5	0	3	1	0	28.9
≡FeOHCa^+3/2^	1	0	0.32	1.68	0	0	0	1	−0.89
≡FeOHCaOH^+1/2^	1	0	0.31	0.69	0	−1	0	1	−6.42
≡FeOH^−1/2^...Ca^+2^	1	0	0	2	0	0	0	1	1.8

ΔZ_0_, ΔZ_1_, and ΔZ_2_ represent the change in the charge in the 0-, 1-, and 2-planes, respectively.

**Table 2:** The As/Fe and Ca/Fe atomic ratio before and after bioreduction obtained by SEM mapping.

Mineral	Experiment Treaments		Before Bioreduction		After Bioreduction	
	As/Fe	Ca/Fe	As/Fe	Ca/Fe	As/Fe	Ca/Fe
Fh	-	-	-	-	-	-
As-Fh	0.2	-	0.104	-	0.207	-
As-Ca-Fh	0.2	0.5	0.169	0.128	0.157	0.017

## Data Availability

Not applicable.

## References

[1] Bhowmick S., Pramanik S., Singh P., Mondal P., Chatterjee D., Nriagu J. (2018). Arsenic in groundwater of West Bengal, India: A review of human health risks and assessment of possible intervention options. Sci. Total Environ..

[2] Palma-Lara I., Martínez-Castillo M., Quintana-Pérez J., Arellano-Mendoza M., Tamay-Cach F., Valenzuela-Limón O., García-Montalvo E., Hernández-Zavala A. (2020). Arsenic exposure: A public health problem leading to several cancers. Regul. Toxicol. Pharmacol..

[3] Zhang Y., Xu B., Guo Z., Han J., Li H., Jin L., Chen F., Xiong Y. (2019). Human health risk assessment of groundwater arsenic contamination in Jinghui irrigation district, China. J. Environ. Manag..

[4] Fendorf S., Michael H.A., van Geen A. (2010). Spatial and temporal variations of groundwater arsenic in South and Southeast Asia. Science.

[5] Muehe E.M., Kappler A. (2014). Arsenic mobility and toxicity in South and South-east Asia–a review on biogeochemistry, health and socio-economic effects, remediation and risk predictions. Environ. Chem..

[6] Abiye T.A., Bhattacharya P. (2019). Arsenic concentration in groundwater: Archetypal study from South Africa. Groundwater. Sustain. Dev..

[7] Cismasu A.C., Michel F.M., Tcaciuc A.P., Tyliszczak T., Brown G.E. (2011). Composition and structural aspects of naturally occurring ferrihydrite. Comptes Rendus Geosci..

[8] Xue Q., Ran Y., Tan Y., Peacock C.L., Du H. (2019). Arsenite and arsenate binding to ferrihydrite organo-mineral coprecipitate: Implications for arsenic mobility and fate in natural environments. Chemosphere.

[9] Tian Z., Feng Y., Guan Y., Shao B., Zhang Y., Wu D. (2017). Opposite effects of dissolved oxygen on the removal of As (III) and As (V) by carbonate structural Fe (II). Sci. Rep..

[10] Coker V., Gault A., Pearce C., Van der Laan G., Telling N., Charnock J., Polya D., Lloyd J. (2006). XAS and XMCD evidence for species-dependent partitioning of arsenic during microbial reduction of ferrihydrite to magnetite. Environ. Sci. Technol..

[11] Muehe E.M., Morin G., Scheer L., Le Pape P., Esteve I., Daus B., Kappler A. (2016). Arsenic (V) incorporation in vivianite during microbial reduction of arsenic (V)-bearing biogenic Fe (III)(oxyhydr) oxides. Environ. Sci. Technol..

[12] Schwertmann U., Stanjek H., Becher H.-H. (2004). Long-term in vitro transformation of 2-line ferrihydrite to goethite/hematite at 4, 10, 15 and 25 °C. Clay Miner..

[13] Zobrist J., Dowdle P.R., Davis J.A., Oremland R.S. (2000). Mobilization of arsenite by dissimilatory reduction of adsorbed arsenate. Environ. Sci. Technol..

[14] Shi W., Wu W., Zeng X., Chen X., Zhu X., Cheng S. (2018). Dissimilatory arsenate-respiring prokaryotes catalyze the dissolution, reduction and release of arsenic from paddy soils into groundwater: Implication for the effect of sulfate. Ecotoxicology.

[15] Tsuchiya T., Ehara A., Kasahara Y., Hamamura N., Amachi S. (2019). Expression of genes and proteins involved in arsenic respiration and resistance in dissimilatory arsenate-reducing Geobacter sp. strain OR-1. Appl. Environ..

[16] Wang L., Cho D.-W., Tsang D.C., Cao X., Hou D., Shen Z., Alessi D., Ok Y.S., Poon C.S. (2019). Green remediation of As and Pb contaminated soil using cement-free clay-based stabilization/solidification. Environ. Int..

[17] Cai X., ThomasArrigo L.K., Fang X., Bouchet S., Cui Y., Kretzschmar R. (2020). Impact of Organic Matter on Microbially-Mediated Reduction and Mobilization of Arsenic and Iron in Arsenic(V)-Bearing Ferrihydrite. Environ. Sci. Technol..

[18] Kocar B.D., Herbel M.J., Tufano K.J., Fendorf S. (2006). Contrasting effects of dissimilatory iron (III) and arsenic (V) reduction on arsenic retention and transport. Environ. Sci. Technol..

[19] Guo H., Wen D., Liu Z., Jia Y., Guo Q. (2014). A review of high arsenic groundwater in Mainland and Taiwan, China: Distribution, characteristics and geochemical processes. Appl. Geochem..

[20] Vital M., Martinez D.E., Babay P., Quiroga S., Clément A., Daval D. (2019). Control of the mobilization of arsenic and other natural pollutants in groundwater by calcium carbonate concretions in the Pampean Aquifer, southeast of the Buenos Aires province, Argentina. Sci. Total Environ..

[21] Yokoyama Y., Mitsunobu S., Tanaka K., Itai T., Takahashi Y. (2009). A study on the coprecipitation of arsenite and arsenate into calcite coupled with the determination of oxidation states of arsenic both in calcite and water. Chem. Lett..

[22] Tamayo-Figueroa D.P., Castillo E., Brandão P.F. (2019). Metal and metalloid immobilization by microbiologically induced carbonates precipitation. World J. Microbiol. Biotechnol..

[23] Beauvois A., Vantelon D., Jestin J., Bouhnik-Le Coz M., Catrouillet C., Briois V., Briois T., Davranche M. (2021). How crucial is the impact of calcium on the reactivity of iron-organic matter aggregates? Insights from arsenic. J. Hazard. Mater..

[24] Alvarez M., Horst M.F., Sileo E.E., Rueda E.H. (2012). Effect of Cd(II) on the ripening of ferrihydrite in alkaline media. Clays Clay Miner..

[25] Alvarez M., Sileo E.E., Rueda E.H. (2005). Effect of Mn(II) incorporation on the transformation of ferrihydrite to goethite. Chem. Geol..

[26] Masue-Slowey Y., Loeppert R.H., Fendorf S. (2011). Alteration of ferrihydrite reductive dissolution and transformation by adsorbed As and structural Al: Implications for As retention. Geochim. Cosmochim. Acta.

[27] Sakakibara M., Tanaka M., Takahashi Y., Murakami T. (2019). Redistribution of Zn during transformation of ferrihydrite: Effects of initial Zn concentration. Chem. Geol..

[28] Liu T., Li X., Waite T.D. (2014). Depassivation of aged Fe0 by divalent cations: Correlation between contaminant degradation and surface complexation constants. Environ. Sci. Technol..

[29] van Genuchten C.M., Gadgil A.J., Peña J. (2014). Fe (III) nucleation in the presence of bivalent cations and oxyanions leads to subnanoscale 7 Å polymers. Environ. Sci. Technol..

[30] Antelo J., Arce F., Fiol S. (2015). Arsenate and phosphate adsorption on ferrihydrite nanoparticles. Synergetic interaction with Ca^2+^. Chem. Geol..

[31] Yuan Z., Zhang G., Lin J., Zeng X., Ma X., Wang X., Wang S., Jia Y. (2019). The stability of Fe (III)-As (V) co-precipitate in the presence of ascorbic acid: Effect of pH and Fe/As molar ratio. Chemosphere.

[32] Cummings D.E., Caccavo F., Fendorf S., Rosenzweig R.F. (1999). Arsenic mobilization by the dissimilatory Fe(III)-reducing bacterium Shewanella alga BrY. Environ. Sci. Technol..

[33] Xiao W., Jones A.M., Li X., Collins R.N., Waite T.D. (2018). Effect of Shewanella oneidensis on the kinetics of Fe (II)-catalyzed transformation of ferrihydrite to crystalline iron oxides. Environ. Sci. Technol..

[34] Stookey L.L. (1970). Ferrozine—A new spectrophotometric reagent for iron. Anal. Chem..

[35] Le X.C., Yalcin S., Ma M. (2000). Speciation of submicrogram per liter levels of arsenic in water: On-site species separation integrated with sample collection. Environ. Sci. Technol..

[36] Zhang D., Wang S., Wang Y., Gomez M.A., Duan Y., Jia Y. (2018). The transformation of two-line ferrihydrite into crystalline products: Effect of pH and media (sulfate versus nitrate). ACS Earth Space Chem..

[37] Tiberg C., Sjöstedt C., Persson I., Gustafsson J.P. (2013). Phosphate effects on copper (II) and lead (II) sorption to ferrihydrite. Geochim. Cosmochim. Acta..

[38] Hiemstra T. (2010). Surface Complexation at Mineral Interfaces: Multisite and Charge Distribution Approach.

[39] Ye C., Ariya P.A., Fu F., Yu G., Tang B. (2021). Influence of Al (III) and Sb (V) on the transformation of ferrihydrite nanoparticles: Interaction among ferrihydrite, coprecipitated Al (III) and Sb (V). J. Hazard. Mater..

[40] Wang Z., Xiao D., Bush R.T., Liu J. (2015). Coprecipitated arsenate inhibits thermal transformation of 2-line ferrihydrite: Implications for long-term stability of ferrihydrite. Chemosphere.

[41] Zhong D., Zhao Z., Jiang Y., Yang X., Wang L., Chen J., Guan C.-Y., Zhang Y., Tsang D.C.W., Crittenden J.C. (2020). Contrasting abiotic As (III) immobilization by undissolved and dissolved fractions of biochar in Ca^2+^-rich groundwater under anoxic conditions. Water Res..

[42] Kanematsu M., Young T.M., Fukushi K., Green P.G., Darby J.L. (2013). Arsenic(III, V) adsorption on a goethite-based adsorbent in the presence of major co-existing ions: Modeling competitive adsorption consistent with spectroscopic and molecular evidence. Geochim. Cosmochim. Acta.

[43] Rietra R.P., Hiemstra T., van Riemsdijk W.H. (2001). Interaction between calcium and phosphate adsorption on goethite. Environ. Sci. Technol..

[44] Liu C., Zhu Z., Li F., Liu T., Liao C., Lee J.-J., Shih K., Tao L., Wu Y. (2016). Fe (II)-induced phase transformation of ferrihydrite: The inhibition effects and stabilization of divalent metal cations. Chem. Geol..

[45] Wang X., Li W., Harrington R., Liu F., Parise J.B., Feng X., Sparks D.L. (2013). Effect of ferrihydrite crystallite size on phosphate adsorption reactivity. Environ. Sci. Technol..

[46] Zhang H., Elskens M., Chen G., Chou L. (2019). Phosphate adsorption on hydrous ferric oxide (HFO) at different salinities and pHs. Chemosphere.

[47] Antelo J., Fiol S., Pérez C., Mariño S., Arce F., Gondar D., López R. (2010). Analysis of phosphate adsorption onto ferrihydrite using the CD-MUSIC model. J. Colloid Interface Sci..

[48] Camacho J.G. (2006). The Influence of Calcium on the Inhibition of Arsenic Desorption from Treatment Residuals in Extreme Environments. https://hdl.handle.net/1969.1/3198.

[49] Stachowicz M., Hiemstra T., van Riemsdijk W.H. (2008). Multi-competitive interaction of As (III) and As (V) oxyanions with Ca^2+^, Mg^2+^, PO^3−^_4_, and CO^2−^_3_ ions on goethite. J. Colloid Interface Sci..

[50] Antelo J., Avena M., Fiol S., López R., Arce F. (2005). Effects of pH and ionic strength on the adsorption of phosphate and arsenate at the goethite–water interface. J. Colloid Interface Sci..

[51] Campbell K.M., Malasarn D., Saltikov C.W., Newman D.K., Hering J.G. (2006). Simultaneous microbial reduction of iron(III) and arsenic (V) in suspensions of hydrous ferric oxide. Environ. Sci. Technol..

[52] Borch T., Masue Y., Kukkadapu R.K., Fendorf S. (2007). Phosphate imposed limitations on biological reduction and alteration of ferrihydrite. Environ. Sci. Technol..

[53] Carabante I., Grahn M., Holmgren A., Hedlund J. (2010). In situ ATR–FTIR studies on the competitive adsorption of arsenate and phosphate on ferrihydrite. J. Colloid Interface Sci..

[54] Reyes C., Murphy J.N., Saltikov C.W. (2010). Mutational and gene expression analysis of mtrDEF, omcA and mtrCAB during arsenate and iron reduction in Shewanella sp. ANA-3. Environ. Microbiol..

[55] Daenzer R., Xu L., Doerfelt C., Jia Y., Demopoulos G.P. (2014). Precipitation behaviour of As(V) during neutralization of acidic Fe(II)−As(V) solutions in batch and continuous modes. Hydrometallurgy.

[56] Tian L., Shi Z., Lu Y., Dohnalkova A.C., Lin Z., Dang Z. (2017). Kinetics of cation and oxyanion adsorption and desorption on ferrihydrite: Roles of ferrihydrite binding sites and a unified model. Environ. Sci. Technol..

[57] Hansel C., Learman D., Lentini C., Ekstrom E. (2011). Effect of adsorbed and substituted Al on Fe(II)-induced mineralization pathways of ferrihydrite. Geochim. Cosmochim. Acta.

[58] Abraham N., James J., Banerji T., Menon R. (2020). Development of a novel groundwater iron removal system using adsorptive Fe(II) process. Groundwater. Sustain. Dev..

[59] Moed D.H., Van Halem D., Verberk J., Amy G.L., Van Dijk J.C. (2012). Influence of groundwater composition on subsurface iron and arsenic removal. Water Sci. Technol..

[60] Muehe E.M., Scheer L., Daus B., Kappler A. (2013). Fate of arsenic during microbial reduction of biogenic versus abiogenic As–Fe (III)–mineral coprecipitates. Environ. Sci. Technol..

[61] Hiemstra T., Van Riemsdijk W.H. (2006). On the relationship between charge distribution, surface hydration, and the structure of the interface of metal hydroxides. J. Colloid Interface Sci..

[62] Huang J.H. (2018). Characterising microbial reduction of arsenate sorbed to ferrihydrite and its concurrence with iron reduction. Chemosphere.

[63] Shi Z., Hu S., Lin J., Liu T., Li X., Li F. (2020). Quantifying microbially mediated kinetics of ferrihydrite transformation and arsenic reduction: Role of the arsenate-reducing gene expression pattern. Environ. Sci. Technol..

